# The net benefit of thrombolysis in the management of intermediate risk pulmonary embolism: Systematic review and meta‐analysis

**DOI:** 10.1002/jha2.97

**Published:** 2020-09-03

**Authors:** Pedro E. Alcedo, Herney Andrés García‐Perdomo, Cristhiam M. Rojas‐Hernandez

**Affiliations:** ^1^ Internal Medicine Department The University of Texas McGovern Medical School Houston Texas; ^2^ Department of Surgery/Urology Universidad del Valle School of Medicine Cali Colombia; ^3^ Section of Benign Hematology The University of Texas M.D. Anderson Cancer Center Houston Texas

**Keywords:** anticoagulation, bleeding, intermediate risk, pulmonary embolism, thrombolysis

## Abstract

**Background:**

Benefit of thrombolytic therapy in patients with massive pulmonary embolism (PE) is evident. However, evidence supporting benefit in clinical outcomes of this approach in intermediate risk PE is lacking.

**Objective:**

To determine the impact of thrombolysis on overall survival in intermediate risk PE patients.

**Methods:**

We searched in MEDLINE (OVID), EMBASE, LILACS, and the Cochrane Central Register of Controlled Trials (CENTRAL) from present day. We also searched in other databases and unpublished literature. We included clinical trials without language restrictions. The risk of bias was evaluated with the Cochrane Collaboration's tool. The primary outcome was overall survival. Secondary outcomes were adverse events, including major bleeding, and all‐cause mortality. The measure of the effect was the risk ratio with a 95% confidence interval (CI).

**Results:**

We included 11 studies in the qualitative and quantitative analysis, with a total of 1855 patients. Risk of bias was variable among the study items. There were no results reported about overall survival in any of the studies. The risk ratio (RR) for all‐cause mortality was 0.68 95% CI (0.40 to 1.16). The RR of overall bleeding, major bleeding and stroke were 2.72 95% CI (1.58 to 4.69), 2.17 95% CI (1.03 to 4.55), and 2.22 95% CI (0.17 to 28.73), respectively. Additionally, the RR for recurrent PE was 0.56 95% CI (0.23 to 1.37).

**Conclusions:**

In patients with intermediate risk PE, the risk of bleeding is higher when thrombolysis is used. There was no significant difference between thrombolysis and anticoagulation in recurrence of PE, stroke, and all‐cause mortality.

## 1 INTRODUCTION

Pulmonary embolism (PE) is a condition that affects approximately 50 per 100 000 adults [1], although an increase in incidence has been reported with improvement in diagnostic modalities [2]. Risk factors can be inherited (eg, hereditary thrombophilias) or acquired (eg, surgery, trauma, cancer) [1, 3]. The morbidity and mortality is typically higher in patients with multiple comorbidities, especially those with cardiopulmonary reserve impairment [4].

Different classifications of PE have been described, according to the anatomic location (saddle, lobar, segmental, subsegmental), the time of presentation (acute, subacute, chronic), or the severity. Massive or high‐risk PE refers to acute PE with hemodynamic instability (systolic blood pressure <90 mmHg), while hemodynamically stable patients are classified as low‐risk PE. Submassive or intermediate‐risk PE is defined as acute PE without systemic hypotension (systolic blood pressure >90 mm Hg) but with either right ventricle (RV) dysfunction or myocardial necrosis [5]. Detection of this subgroup of patients is important, since the presence of RV dysfunction predicts worse clinical outcome [6, 7, 8, 9, 10, 11].

A variety of therapeutic strategies have been evaluated for the acute management of high‐risk situations, and they include predominantly thrombolytic procedures. Thrombolysis has proven to be a treatment modality with survival benefit in patients with massive PE [12]; however, the risk: benefit ratio is less clear in cases that present without hypotension [13]. In intermediate risk PE, there are data supporting the use of thrombolytic procedures to improve hemodynamic parameters, although the mortality benefit of these interventions in the light of the risk of bleeding remains uncertain [14].

The current standard of care for the management of acute PE with hemodynamic stability is systemic anticoagulation, and duration of treatment varies based on the presence of risks factors and the overall risk of bleeding in patient‐specific populations [15]. The recommendation for the use of thrombolytic therapy is limited to acute PE with hypotension after excluding major contraindications associated with a higher risk of bleeding (intracranial metastases, thrombocytopenia, recent bleeding, etc.) [5, 16].

There is no homogeneous consensus among different guidelines on the management of patients with intermediate risk PE [15, 17]. The objective of this meta‐analysis was to determine the clinical benefits and harms of thrombolysis followed by anticoagulation versus anticoagulation alone, in patients with intermediate risk PE.

## 2 METHODS

We performed this review according to the recommendations of the Cochrane Collaboration and following the PRISMA Statement. The PROSPERO registration number is CRD42019128229.

### 2.1 Eligibility criteria

Clinical trials involving adults with submassive or intermediate risk PE, as previously defined, were included. The intervention was thrombolytic therapy compared to systemic anticoagulation in the control group. Studies that involved patients with a clear indication for thrombolysis (massive PE), and studies where both intervention and control groups received different doses of thrombolytic agents were excluded. There were no setting or language restrictions.

### 2.2 Outcomes

Primary outcome was overall survival. Secondary outcomes were adverse events, including major bleeding and stroke, changes in parameters of RV strain, and all‐cause mortality. Major bleeding was defined according to the International Society of Thrombosis and Haemostasis [18], or the criteria used by the authors in the original studies if information was not available.

For all outcomes, studies had at least 4 weeks follow‐up and the outcomes were assessed at different time points regarding the studies included.

### 2.3 Information sources

Literature search was conducted in accordance to recommendations by Cochrane. We used medical subject headings (MeSh), Emtree language, Decs and text words related. We searched MEDLINE (OVID), EMBASE, LILACS, and the Cochrane Central Register of Controlled Trials (CENTRAL) from inception to nowadays (Appendix 1). To ensure literature saturation, we scanned references from relevant articles identified through the search, conferences, thesis databases, Open Gray database, Google scholar, and clinicaltrials.gov, among others. We contacted authors by e‐mail in case of missing information.

### 2.4 Data collection

Each reference by title and abstract was reviewed by three researchers. Then, full‐text of relevant studies were scanned for data extraction, applying prespecified inclusion, and exclusion criteria. Disagreements were resolved by consensus among the three authors. Using a standardized form, one reviewer extracted the following information from each article: study design, geographic location, author's names, title, objective, inclusion and exclusion criteria, number of patients included, losses to follow‐up, timing, definition of outcomes, outcomes and association measures, and funding source.

### 2.5 Risk of bias

For clinical trials, the Cochrane Collaboration tool was utilized, which covers sequence generation, allocation concealment, blinding, incomplete outcome data, selective reporting, and other biases. Researchers judged about the possible risk of bias from the extracted information, rating it as “high risk,” “low risk,” or “unclear risk,” Graphic representation of potential bias was computed using Review Manager 5.3 (RevMan^®^ 5.3).

### 2.6 Data analysis/synthesis of results

The statistical analysis was performed using RevMan^®^ 5.3. For categorical outcomes, information about risk ratios (RR) with 95% confidence intervals was reported, and the information was pooled with a random effect meta‐analysis according to the heterogeneity expected. The results were reported in forest plots of the estimated effect of the included studies with a 95% confidence interval (95% CI). Heterogeneity was evaluated using the *I* ^2^ test. For the interpretation, it was determined that values of 25%, 50%, and 75% correspond to low, medium, and high levels of heterogeneity, respectively.

### 2.7 Publication bias

Potential publication bias was assessed using a funnel plot for all‐cause mortality and overall bleeding since these were the outcomes evaluated by more than 10 studies.

### 2.8 Sensitivity analysis

We performed sensitivity analysis extracting weighted studies and running the estimated effect to find differences.

### 2.9 Subgroup analysis

We performed a subgroup analysis based on the specific type of thrombolytic used (r‐TPA vs nonrecombinant TPA).

## 3 RESULTS

### 3.1 Study selection

**FIGURE 1:**
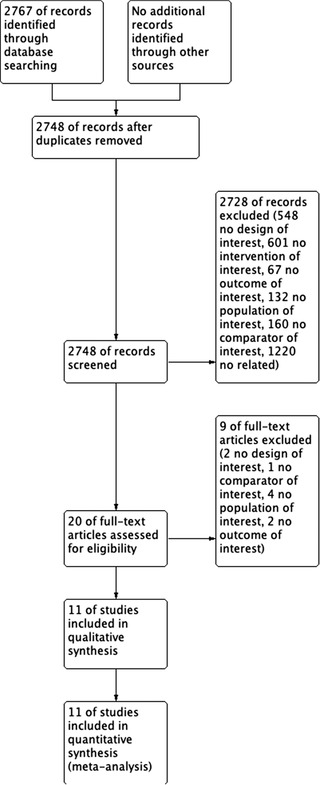
Flowchart of included studies

After an extensive review of the literature, we found 2767 records with the designed search strategies. Finally, 11 studies were included in the qualitative and quantitative analysis [19, 20, 21, 22, 23, 24, 25, 26, 27, 28, 29] (Figure 1).

### 3.2 Included studies

We included a total of 1855 patients, with a mean of 167 patients per study. All studies presented information about all‐cause mortality [19, 20, 21, 22, 23, 24, 25, 26, 27, 28, 29]. Regarding the adverse events, eight studies showed information about major bleeding [19, 20, 22–25, 27, 29]; 11 about overall bleeding [19–23, 25–28]; two about stroke [25, 27]; and six about recurrent PE [19, 20, 23, 25, 27, 28] (Table 1).

**TABLE 1:** Characteristics of included studies

First author	Country	Methods	Outcomes	Interventions	Follow‐up
Goldhaber et al., 1993^23^	USA	Separate, nonblinded, open label treatment assignments for each hospital were generated by permuted block random number sequences. Data were analyzed by randomization assignment (intention to treat). A total of 101 patient were included	RVD (echocardiogram), pulmonary tissue perfusion (VQ scan), recurrent PE	Alteplase + UFH (*n* = 46) vs UFH (*n* = 55)	21 days
Becattini et al., 2010^19^	Italy	Double blind, placebo controlled study. A total of 58 patients were randomized in a 1:1 ratio, generated in blocks of four. Allocation to treatment performed based on progressive treatment number.	Reduction of RVD (echocardiogram), clinical deterioration requiring escalation of treatment, recurrent PE (by image modality or sudden death), death at 30 days from randomization	Tenecteplase + UFH (*n* = 28) vs placebo + UFH (*n* = 30)	30 days
Meyer et al., 2014^27^	Germany	Double blind, placebo controlled. A total of 1006 patients underwent central randomization with the use of a computerized Internet‐based system. Randomization was stratified by center and, within centers, was performed in blocks. The main efficacy and safety analyses were based on all events that occurred in the intention‐to‐treat population.	Death from any cause within 7 and 30 days of randomization, hemodynamic decompensation, bleeding, stroke, recurrent PE	Tenecteplase + UFH (*n* = 506) vs placebo + UFH (*n* = 500)	30 days
Konstantinides et al., 2002^25^	Germany	Double blind, placebo controlled. Randomization was performed on a 1:1 basis with a fixed block size of six patients at each center; a total of 256 patients were randomized. Statistical analysis was performed according to the intention‐to‐treat principle.	Death or clinical deterioration requiring escalation of treatment, recurrent PE (confirmed by imaging study), major bleeding, ischemic stroke	Alteplase + UFH (*n* = 118) vs placebo + UFH (*n* = 138)	30 days
Kucher et al., 2014^26^	Switzerland	Patient that met criteria were randomized in an open‐label fashion. Randomization of 59 patients was performed in four blocks without stratification.	Difference in RV/LV ratio at 24 hours of treatment by echocardiogram. Death, hemodynamic decompensation, bleeding, recurrent VTE (confirmed by an imaging test), serious adverse events.	CDT rtPA + UFH (*n* = 30) vs UFH (*n* = 29)	90 days
Sharifi et al., 2013^28^	USA	Controlled, randomized, single‐center open study. 121 patients were included	Pulmonary hypertension (by echocardiography); composite of pulmonary hypertension and recurrent PE. Total mortality, length of hospital stay, bleeding, composite of mortality, and PE recurrence	tPA + UFH or LMWH (n = 61) vs UFH or LMWH (n = 60)	Mean 28 ± 5 months
Fasullo et al., 2011^22^	Italy	Double blind, placebo controlled. Randomization of 72 patients was performed by using a preliminary computer algorithm. Assignment of all patients was decided on admission, before obtaining imaging studies, by an external team of physicians.	Reduction of RVD (by echocardiogram). Recurrent PE (by image modality or sudden death), death during hospitalization and at 180 days from randomization, clinical events, major bleeding.	Alteplase + UFH (*n* = 37) vs placebo + UFH (*n* = 35)	6 months
Taherkhani et al., 2014^21^	Iran	Single blind study. Eligible patients underwent randomization with the use of a computerized system, and randomization was performed in blocks. Fifty patients were included.	Death, clinical deterioration requiring escalation of treatment. Major bleeding, ischemic stroke, pulmonary hypertension and RV dilation (by echocardiogram), dyspnea.	Alteplase or streptokinase + enoxaparin (*n* = 25) vs enoxaparin (n = 25)	30 days
Kline et al., 2014^24^	USA	Double blind, placebo controlled, intention‐to‐treat. Study group assignment occurred by a predetermined, blocked permuted 1:1 randomization sequence. Eighty‐three patients were included.	Death (from PE, hemorrhage), shock, need for intubation, bleeding, VTE recurrence (image proven), functional capacity, quality of life (SF 36 score).	Tenecteplase + LMWH (*n* = 40) vs placebo + LMWH (n = 43)	90 days
Dalla‐Volta et al., 1992^20^	Italy	Open, parallel, randomized trial. Thirty‐six patient included.	Pulmonary angiographic index (Miller index)	Alteplase + UFH (*n* = 20) vs UFH (*n* = 16)	30 days
Stein et al., 1990^29^		Double blind randomization. 13 patients included	Vessel occlusion by pulmonary arteriogram, perfusion defects by V/Q scan, hemodynamic changes, evidence of fibrinolysis (D‐dimer)	rt‐PA + heparin (*n* = 9) vs placebo + heparin (*n* = 4)	

Abbreviations: CDT: catheter‐directed thrombolysis; LMWH: low molecular weight heparin; LV: left ventricle; PE: pulmonary embolism; rtPA: recombinant tissue plasminogen activator; RV: right ventricle; RVD: right ventricle dysfunction; UFH: unfractioned heparin; VTE: venous thromboembolism.

### 3.3 Risk of bias

Most studies had low risk of bias for random sequence generation, blinding of outcome assessment, selective reporting, and other bias. On the other side, regarding the blinding of participants and personnel, incomplete outcome data, and allocation concealment issues, there was unclear and high‐risk bias for most of the studies (Figure 2A and 2B).

FIGURE 2(A) Risk of bias within studies. (B) Risk of bias among studies

**Figure:**
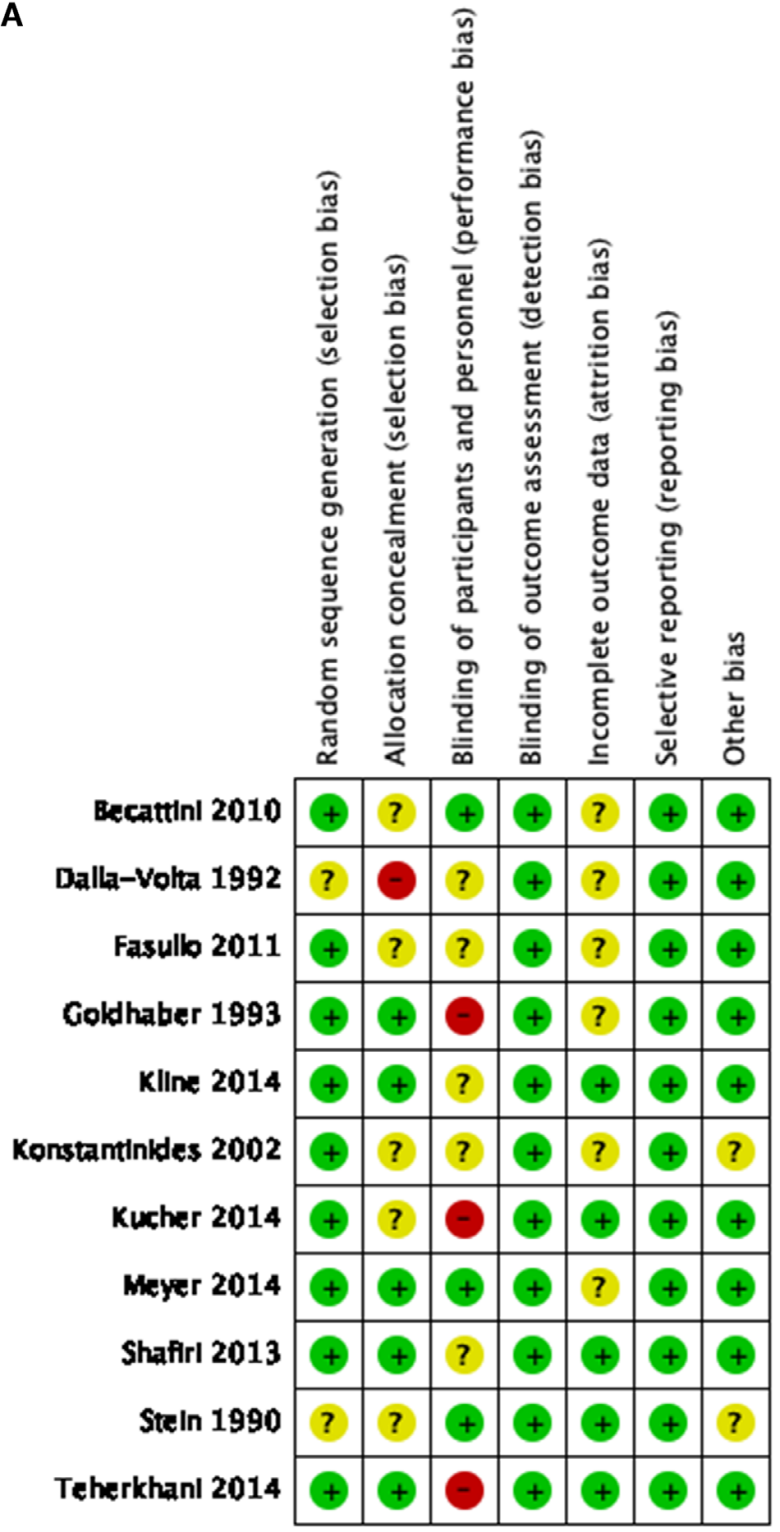

**Figure:**
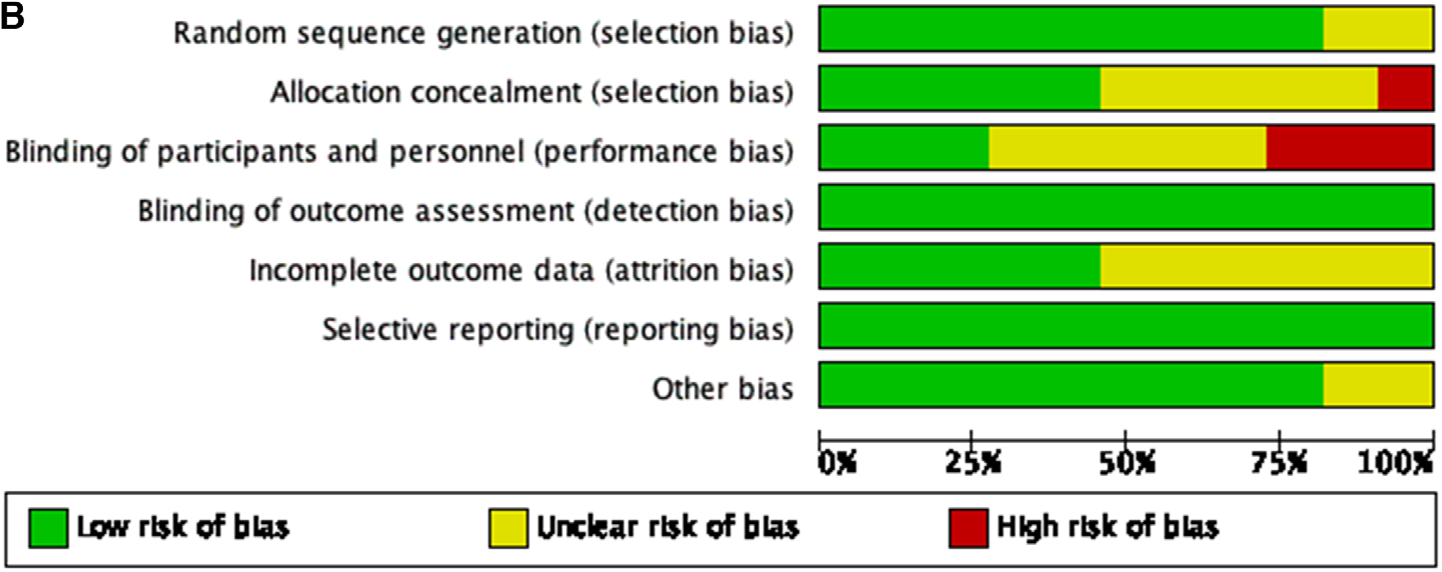

### 3.4 Primary outcome: overall survival

There were no results reported about overall survival among the included studies.

### 3.5 Secondary outcomes

Secondary outcomes were adverse events, including major bleeding, changes in parameters of RV strain, and all‐cause mortality.

When evaluating for major bleeding, results from eight studies also favored the anticoagulation only‐intervention group RR 2.17 (1.03 to 4.55) *I* ^2 ^= 26% (Figure 3A). Regarding overall bleeding risk, data from 11 studies showed a difference favoring the anticoagulation only‐intervention group RR 2.72 95% CI (1.58 to 4.69) *I* ^2 ^= 26% (Figure 3B). Six studies reported results on recurrent PE suggesting benefit from thrombolysis, but this difference was not statistically significant, RR 0.56 (0.23 to 1.37) *I* ^2 ^= 0% (Figure 3C). Only two studies included outcomes on risk of stroke and found no advantage of thrombolysis over anticoagulation‐only intervention, RR 2.22 (0.17 to 28.73) *I* ^2 ^= 56% (Figure 3D).

FIGURE 3(A) Meta‐analysis of included studies. Comparison: systemic anticoagulation only (SA) versus thrombolytic therapy (TT). Outcome: major bleeding. (B) Meta‐analysis of included studies. Comparison: SA versus TT. Outcome: overall bleeding. (C) Meta‐analysis of included studies. Comparison: SA versus TT. Outcome: recurrent pulmonary embolism (PE). (D) Meta‐analysis of included studies. Comparison: SA versus TT. Outcome: stroke. (E) Meta‐analysis of included studies. Comparison: SA versus TT. Outcome: all‐cause mortality

**Figure:**
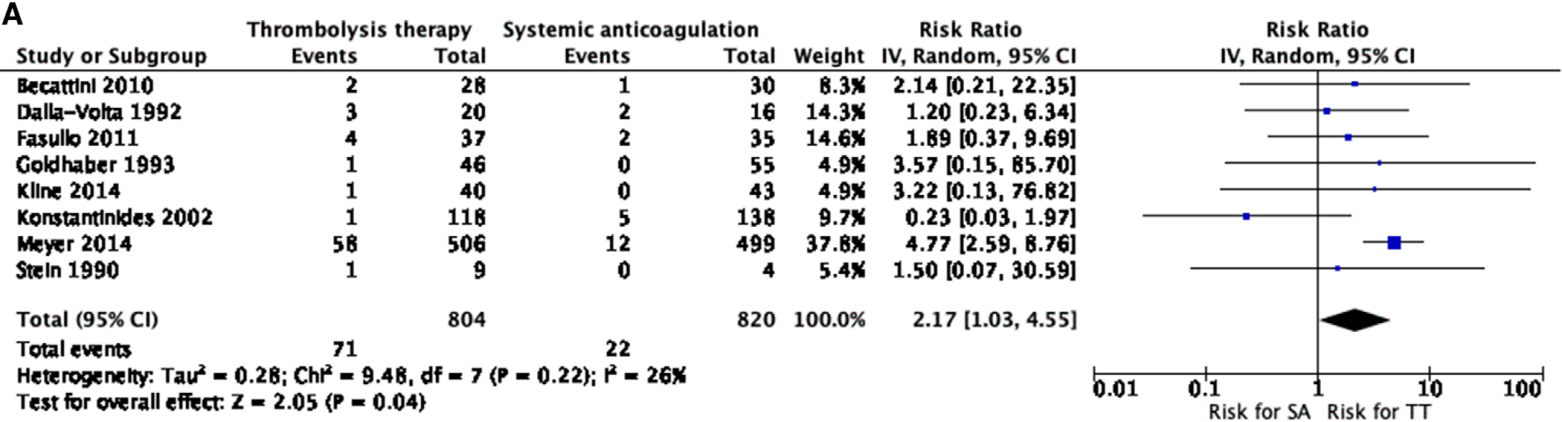

**Figure:**
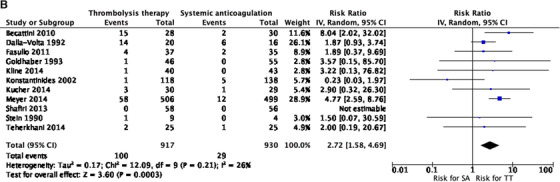

**Figure:**
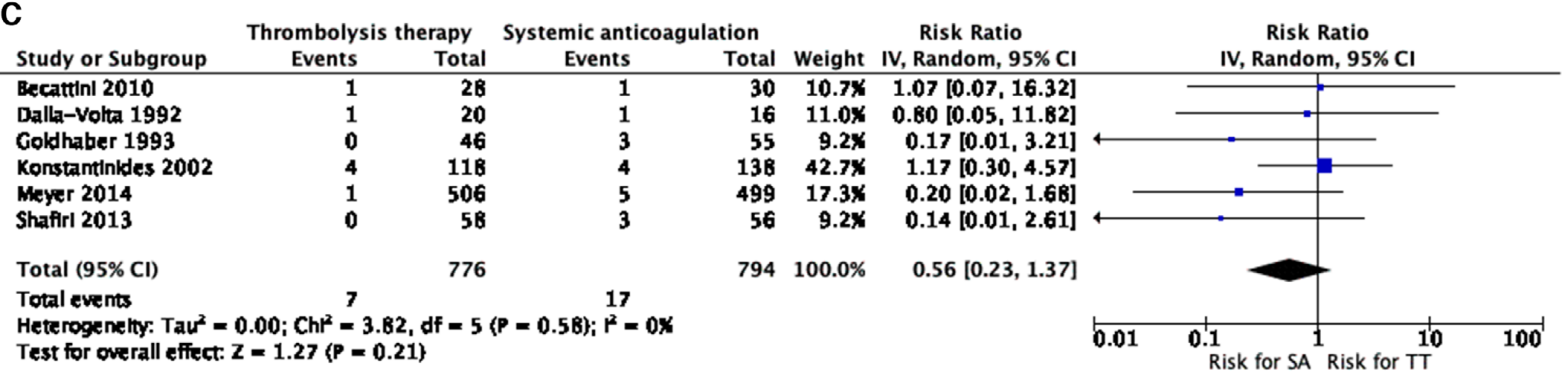

**Figure:**
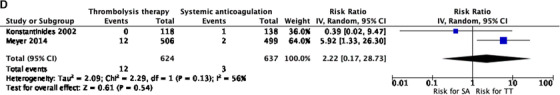

**Figure:**
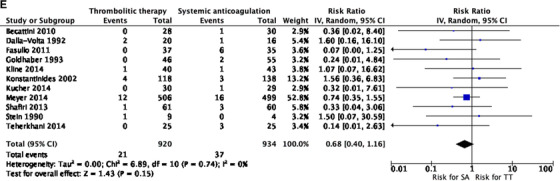

Analysis of the impact different therapies had in RV compromise was not feasible, since there was substantial heterogeneity in the variables used among studies (RV wall motion, septum dynamics, right/left ventricle end‐diastolic dimension ratio) and in the methods used for evaluation (electrocardiogram, echocardiogram, pulmonary artery hemodynamics, markers of cardiac injury).

All‐cause mortality was evaluated in all studies, favoring the group that received thrombolysis, yet difference was not statistically significant, RR 0.68 (0.40 to 1.16) *I* ^2 ^= 0% (Figure 3E).

### 3.6 Publication bias

We did not find any publication bias (Supplementary files 1 and 2).

### 3.7 Sensitivity analysis

When excluding the study by Meyer et al [27] for the outcome major bleeding, we found a RR of 1.33 95% CI (0.58 to 3.05). There were no differences between the two interventions; therefore, Meyer et al. influenced on the final effect.

Regarding the outcome overall bleeding, we found a RR of 2.37 95% CI (1.06 to 5.29). Therefore, there was no change compared with the effect found in the general analysis.

### 3.8 Subgroup analysis

Analysis of results based on thrombolytic regimen used showed that the risk of overall and major bleeding is higher with thrombolysis, when compared to anticoagulation alone. However, this is statistically significant for tenecteplase, but not for alteplase. Regarding recurrent PE and all‐cause mortality, there was no difference between thrombolysis and anticoagulation alone, regardless of thrombolytic chosen (Table 2). Four studies did not specify which specific thrombolytic agent was used; therefore, they were not included in the subgroup analysis [21, 26, 28, 29].

**TABLE 2:** Summary of clinical outcomes by fibrinolytic agent

Studies	Outcome	Type	Risk ratio	95% confidence interval	*I* ^2^ (%)
Becattini 2010; Kline 2014; Meyer 2014	Major bleeding	Tenecteplase	4.31	2.44 to 7.61	0
Dalta‐Volta 1992; Fasullo 2011; Konstantinides 2002; Goldhaber 1993	Major bleeding	Alteplase	1.11	0.42 to 2.94	0
Becattini 2010; Meyer 2014	Total bleeding	Tenecteplase	5.19	2.97 to 9.06	0
Dalta‐Volta 1992; Fasullo 2011; Konstantinides 2002; Goldhaber 1993	Total bleeding	Alteplase	1.51	0.68 to 3.32	17
Becattini 2010 ;Kline 2014; Meyer 2014	All‐cause mortality	Tenecteplase	0.73	0.36 to 1.47	0
Dalta‐Volta 1992; Fasullo 2011; Konstantinides 2002; Goldhaber 1993	All‐cause mortality	Alteplase	0.66	0.16 to 2.65	33
Becattini 2010; Meyer 2014	Recurrent PE	Tenecteplase	0.38	0.07 to 2.03	0
Dalta‐Volta 1992; Konstantinides 2002; Goldhaber 1993	Recurrent PE	Alteplase	0.83	0.27 to 2.54	0

## 4 DISCUSSION

### 4.1 Summary of the main results

Review of trials comparing thrombolytic therapy versus anticoagulation, specifically including a large sample of patients with intermediate risk PE, showed higher bleeding associated with thrombolysis. There was no difference in all‐cause mortality, PE recurrence, and stroke. Evidence about overall survival is lacking.

### 4.2 Contrast with the literature

The net benefit of thrombolytic strategies in the management of intermediate risk PE continues to be an area of active debate. Based on the results of our meta‐analysis, we agree with other authors that the risk of bleeding is higher in patients who receive thrombolysis [30, 31, 32, 33, 34, 35, 36, 37]. However, our study did not show a benefit on all‐cause mortality, risk of stroke, and recurrence of PE, when thrombolysis was added to anticoagulation.

The PEITHO trial, the largest study that has included patients with intermediate risk PE, showed a clear advantage of thrombolysis in hemodynamic status and need of life‐sustaining measures, yet no difference in 30‐day mortality was found [27]. Although other meta‐analysis have reported an improvement in mortality in the intervention group [32, 35], we believe these results are driven by the composite primary outcome of the PEITHO trial, which includes early death and hemodynamic decompensation; this observation was also made by another group of authors [36]. A most recent analysis of multiple systematic reviews also reported a reduction in mortality secondary to thrombolysis, but recognizes that results are inconsistent, the large use of antinomies, and the questionable quality of some of the studies included [38]. Other factors that can explain the discordant results among studies, include different definitions of intermediate risk PE; criteria and diagnostic methods of RV dysfunction; type, dose, and route of administration of thrombolytic; definition of adverse outcomes.

Previous evidence suggests that benefit of thrombolysis in overall mortality is less clear once studies containing high‐risk PE patients are excluded [30, 33] and after long‐term follow‐up [39]. This emphasizes that PE patient population is heterogeneous, and several factors should be taken into account when choosing the optimal therapeutic regimen for every patient. Data seem to be consistent regarding the particular increased risk of bleeding in older patients, but a precise age cutoff has not been established [27, 31], and risk stratification tools have not been validated and are not routinely used [40].

Optimal thrombolytic agent, dose, and route of administration continue to be a matter of debate; since historically, there is no consistency in treatment regimens used among different studies in PE patients. Authors have described a higher risk of bleeding among different thrombolytic agents, such as tenecteplase [30], which could be related to its higher potency. Our subgroup analysis corroborates this finding, showing a statistically significant higher risk of bleeding when tenecteplase is compared with anticoagulation alone; similarly, subgroup of patients who received alteplase seemed to have higher risk of bleeding, but the difference was not significant.

Safety of thrombolytics has been studied in more detail in patients with myocardial infarction (MI) and stroke, and evidence seems to differ from what studies have shown in PE patients. In acute ST segment elevation MI, tenecteplase has shown similar efficacy and safety profile compared to alteplase, without an increase in bleeding risk [41]. One group even showed that tenecteplase was associated with fewer noncerebral bleeding complications [42]. In stroke trials, clinical outcomes have also been similar between two groups, and tenecteplase has not shown increased risk of bleeding [43, 44, 45, 46, 47]. Studies comparing different thrombolytics among each other, focusing in patients with PE, are needed to clarify this point.

In the past few years, there has been increasing interest in catheter‐directed therapy (CDT) with lower doses of thrombolytics to minimize risk of bleeding while maintaining clinical efficacy. Different doses and infusion duration have shown improvement in parameters of RV function and decreased bleeding risk with this strategy, including patients with intermediate risk PE [48, 49], but impact in mortality and long‐term clinical outcomes need to be evaluated to better understand the benefit of this approach. Studies comparing different devices for CDT, or this technology with systemic fibrinolysis, or anticoagulation are ongoing [39, 50]; hopefully these results will help clarify if thrombolysis is a good option for patients with intermediate risk PE.

### 4.3 Strengths and limitations

The inclusion of patients with intermediate risk PE alone is the main strength of our study. The number of patients included is also significant. Studies included in previous revisions were also included in our meta‐analysis.

Our meta‐analysis has limitations. Definitions of outcomes, particularly major bleeding, and follow‐up times were not consistent among authors. Likewise, thrombolytic agents and doses used are different. Therefore, conclusions in regard to comparison of outcomes related to specific thrombolytic agents, dosage, and protocol of administration were not possible.

Other limitations are related to the high risk of bias associated with the unclear blinding of participants and personnel, incomplete outcome data, and allocation concealment issues of the studies included in the meta‐analysis.

Although the primary objective of this study was to evaluate overall survival, we did not find any data in this specific subgroup of patients with intermediate risk PE. This is one of multiple questions that remain unanswered and requires further studies.

## 5 CONCLUSIONS

In patients with intermediate risk PE, overall and major bleeding risks are higher when thrombolytic therapy is added to anticoagulation. Systemic thrombolytic strategies do not show a statistically significant clinical benefit on recurrence of PE, stroke, and all‐cause mortality, when compared to anticoagulation alone. Efforts to provide safe and effective therapies, minimizing bleeding risk, must continue; current ongoing studies evaluating the role of novel thrombolytic strategies (eg, CDT) may provide a better evidence for the use of thrombolytic treatments in the population of intermediate risk PE.

## CONFLICT OF INTEREST

Dr. Rojas‐Hernandez has received funding for research from Daichii Sankyo Pharmaceuticals and Aspen Pharmaceuticals not related to the content of this manuscript. Dr. Alcedo and Dr Garcia‐Perdomo declare no conflict of interest.

## AUTHORS CONTRIBUTION

All authors contributed substantially to the study design, data analysis and interpretation, and the writing of the manuscript.

## AVAILABILITY OF DATA AND MATERIAL

Data collected for this study will be available upon request.

## Supporting information

Supporting information

Supporting information
