# Supplementary material for: The net benefit of thrombolysis in the management of intermediate risk pulmonary embolism: Systematic review and meta‐analysis
Source: EJHaem. 2020 Sep 3;1(2):457–66. doi: 10.1002/jha2.97 (PMC9176023; doi:10.1002/jha2.97)
Supplement: Supplementary file 1 — Supporting information [file JHA2-1-457-s001.docx]

**Appendix 1. Search Strategy**

**Medline (Ovid):**

(Exp pulmonary embolism or (Pulmonary adj2 infarction).mp or (Submassive adj2 pulmonary adj2 embolism).mp or (pulmonary adj2 thrombosis).mp or (Pulmonary adj2 embolism).mp or (submassive adj2 pulmonary adj2 thrombosis).mp) AND (Exp thrombolytic therapy or Thrombolysis.mp or Fibrinolysis.mp or (Fibrinolytic adj2 agent*).mp or Tissue Plasminogen Activator or Alteplase.mp or Tenecteplase.mp or Urokinase-Type Plasminogen Activator or Urokinase.mp or Streptokinase or Streptokinase.mp) AND (randomized controlled trial.pt or controlled clinical trial.pt or randomized.ab or placebo.ab or randomly.ab or trial.ab or (clinical adj2 trial).mp or (randomi*ed adj2 controlled adj2 trial).mp or exp double-blind method or exp cohort studies or (cohort* adj2 stud*).mp)

**Embase:**

('lung embolism'/exp or (Pulmonary next/2 infarction):ti,ab or (Submassive next/2 pulmonary next/2 embolism):ti,ab or (pulmonary next/2 thrombosis):ti,ab or (Pulmonary next/2 embolism):ti,ab or (submassive next/2 thrombosis):ti,ab) AND ('fibrinolytic therapy'/exp or Thrombolysis:ti,ab or Fibrinolysis:ti,ab or (Fibrinolytic next/2 agent*):ti,ab or 'tissue plasminogen activator'/exp or 'alteplase'/exp or Alteplase:ti,ab or 'tenecteplase'/exp or Tenecteplase:ti,ab or 'urokinase'/exp or Urokinase:ti,ab or 'streptokinase'/exp or Streptokinase:ti,ab) AND ('randomized controlled trial'/exp or (randomi*ed NEXT/2 controlled NEXT/2 trial):ti,ab or 'clinical trial'/exp or (clinical NEXT/2 trial):ti,ab or 'double blind procedure'/exp or 'cohort analysis'/exp or cohort*:ti,ab)

**Central (Ovid)**

(Exp pulmonary embolism or (Pulmonary adj2 infarction).mp or (Submassive adj2 pulmonary adj2 embolism).mp or (pulmonary adj2 thrombosis).mp or (Pulmonary adj2 embolism).mp or (submassive adj2 pulmonary adj2 thrombosis).mp) AND (Exp thrombolytic therapy or Thrombolysis.mp or Fibrinolysis.mp or (Fibrinolytic adj2 agent*).mp or Tissue Plasminogen Activator or Alteplase.mp or Tenecteplase.mp or Urokinase-Type Plasminogen Activator or Urokinase.mp or Streptokinase or Streptokinase.mp)
